# Impact of rising temperatures on historical wheat yield, phenology, and grain size in Catalonia

**DOI:** 10.3389/fpls.2023.1245362

**Published:** 2023-10-11

**Authors:** Davide Gulino, Roser Sayeras, Joan Serra, Josep Betbese, Jordi Doltra, Adrian Gracia-Romero, Marta S. Lopes

**Affiliations:** ^1^ Sustainable Field Crops Program, IRTA (Institute of Agrifood Research and Technology), Lleida, Spain; ^2^ Sustainable Field Crops Program, IRTA (Institute of Agrifood Research and Technology), Girona, Spain

**Keywords:** wheat, yield progress, climate change, genetic gain, temperature

## Abstract

**Introduction:**

Climate change poses significant challenges to agriculture, impacting crop yields and necessitating adaptive strategies in breeding programs. This study investigates the genetic yield progress of wheat varieties in Catalonia, Spain, from 2007 to 2021, and examines the relationship between genetic yield and climate-related factors, such as temperature. Understanding these dynamics is crucial for ensuring the resilience of wheat crops in the face of changing environmental conditions.

**Methods:**

Genetic yield progress was assessed using a linear regression function, comparing the average yield changes of newly released wheat varieties to benchmark varieties. Additionally, a quadratic function was employed to model genetic yield progress in winter wheat (WW). The study also analyzed correlations between genetic yield (GY) and normalized values of hectoliter weight (HLW) and the number of grains (NG) for both spring wheat (SW) and WW. Weather data were used to confirm climate change impacts on temperature and its effects on wheat growth and development.

**Results:**

The study found that genetic yield was stagnant for SW but increased linearly by 1.31% per year for WW. However, the quadratic function indicated a possible plateau in WW genetic yield progress in recent years. Positive correlations were observed between GY and normalized values of HLW and NG for both SW and WW. Climate change was evident in Catalonia, with temperatures increasing at a rate of 0.050 °C per year. This rise in temperature had detrimental effects on days to heading (DH) and HLW, with reductions observed in both SW and WW for each °C increase in annual minimum and average temperature.

**Discussion:**

The findings highlighted the urgent need to address the impact of climate change on wheat cultivation. The stagnation of genetic yield in SW and the potential plateau in WW genetic yield progress call for adaptive measures. Breeding programs should prioritize phenological adjustments, particularly sowing date optimization, to align with the most favorable months of the year. Moreover, efforts should be made to enhance HLW and the number of grains per unit area in new wheat varieties to counteract the negative effects of rising temperatures. This research underscores the importance of ongoing monitoring and adaptation in agricultural practices to ensure yield resilience in the context of a changing climate.

## 1 Introduction

Wheat is the most cultivated crop in the world covering 220.76 million ha, followed by maize (205.87 million ha), and rice (165.25 million ha), and represents a third of the total grain production with an estimated value of 770 million tons, following rice (787 million tons) and maize (1,210 million tons) (FAOSTAT, 2021). In Europe, it is also the most cultivated cereal with 62.82 million ha followed by barley with 22.52 million ha, while in Spain, barley is first with 2.51 million ha followed by wheat with 2.12 million ha (FAOSTAT, 2021). The same pattern was observed in Catalonia, NE of Spain, where this study was performed: barley covers 154.574 ha, as the first cultivated cereal, while wheat covers 103.149 ha (Statistical Institute of Catalonia, 2021). Based on the importance of wheat at both the local and global scales among staple food crops, massive breeding efforts are required to support challenges in food security, considering the consistent increase in the world population. Currently, climate change represents an additional challenge to provide high-yield varieties that may adapt to extreme environmental conditions. Climate change is projected to decrease the global wheat yield by approximately 1.9% by 2050, affecting mostly developing countries, such as in Africa and Southern Asia, where food security is already a problem (Pequeno et al., 2021). Furthermore, the co-occurrence of extremely hot and dry events from 1980 to 2009 had a global negative impact on the yield of major cereal crops, and its probability increased by up to six times in wheat-specific growing regions (Heino et al., 2023). In addition, Asseng et al. (2015) used 30 different wheat crop models to demonstrate a 6% decrease in yield for each degree Celsius increase in most wheat-growing regions. Considering these predictions, periodic evaluation of the rate of genetic gain in grain yield is crucial to estimate how breeding efforts effectively contribute to satisfying the increasing global food demand and to identify new potential avenues for future improvement. Crop yield progress is defined as the slope of the linear regression function between the average yield and time (Sayre et al., 1997), which provides information on the impact of breeding on yield or other traits of interest. Long-term check varieties (the most widely grown in the region) are included every year in post-registration trials, allowing for the estimation of yield gain, which is calculated as the yield percentage of new varieties against the yield of long-term check varieties every year. Thus, it is possible to evaluate the rate of grain yield increase across years in such trials (Graybosch and Peterson, 2010; Crespo-Herrera et al., 2018). Moreover, yield progress can be internally assessed in wheat breeding programs to track the impact of breeding on new varieties. However, to evaluate progress in the yield available to farmers, all new wheat varieties released by the private and public sectors in a certain region over time must be assessed. Using this evaluation, the potential variety portfolio available to farmers in any given year can be evaluated. In this study, progress in yield was assessed using a set of all available varieties between 2007 and 2021 (from all public and private breeding programs). Specifically, this study aimed to (1) determine the extent of genetic yield progress (if any) in the last 15 years (2007–2021) in both spring wheat (SW) and winter wheat (WW) varieties; (2) explain which agronomic traits contribute to yield progress or stagnation using correlation analysis; and (3) evaluate the extent of climate change by analyzing weather trends across time and their impact on yield, yield components, phenology, and other agronomic traits.

## 2 Materials and methods

### 2.1 Experimental data

**Figure 1:**
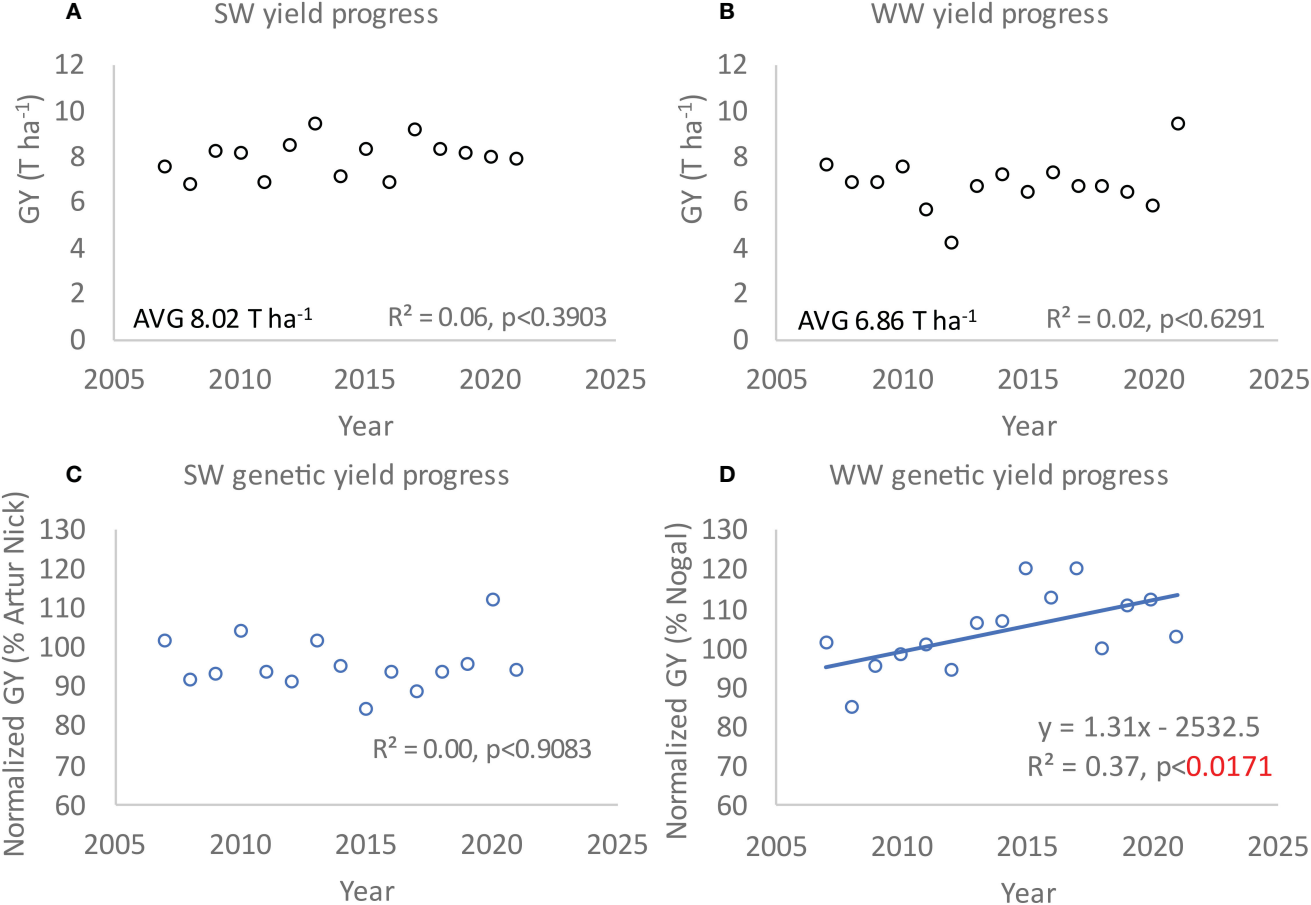
**(A, B)** Spring wheat (SW) and winter wheat (WW) yield progress in Catalonia between 2007 and 2021 in absolute values. **(C, D)** Normalized yield expressed as the percentage of GY variation against a check spring wheat variety “Artur Nick” and a check winter wheat variety “Nogal”. Spring and winter wheat GY was obtained from experimental untreated (no pesticides applied) trials conducted in the regions of La Tallada (rainfed) and Lleida (irrigated), Vic, Solsona, and Vilobí d’Onyar, respectively. Means of all the varieties tested each year in each location are plotted and regression equations are shown with coefficients of determination (*R* ^2^) and associated probability (*p* < 0.05 in red).

The Institute of Agrifood Research and Technology (IRTA) coordinates a field trial network (post-registration variety testing trials, https://extensius.cat/xarxes-de-varietats/) in the Catalonia region to provide farmers with information on the most adapted varieties of various arable crops annually. These trials evaluate an approximate annual average of 20 new SW and 38 WW varieties, regardless of their potential adoption by farmers, as detailed in **Supplementary Table 1**. These evaluations are conducted against established benchmark check varieties (“Artur Nick” for SW and “Nogal” for WW) widely cultivated in the region. The varieties available in the Catalonia market are annually evaluated, reporting data on agronomic traits and adaptation to the various wheat-growing regions. These replicated trials were conducted using experimental micro-plots (8 m × 1.2 m) located in the most representative production areas distributed throughout the different agroclimatic zones of Catalonia. These areas are all characterized by a Mediterranean climate, with hot summers and mild winters. The post-registration variety testing trials for WW were conducted at three rainfed locations representative of areas with cooler winters from West to East counties: Solsona (county Solsonès), Vic (county of Osona), and Vilobí d’Onyar (county of La Selva). For SW, the locations were Lleida (county of Segrià), irrigated trial, and La Tallada d’Empordà (county of Baix Empordà), rainfed, representatives respectively of the West and East warmer wheat production areas of Catalonia. **Table 1** shows the average long-term weather data at each location. The varieties tested in these trials were not treated with pesticides. For each variety and year of trial, agronomic traits were determined using the following methods: grain yield (GY) at 13% humidity was determined by machine harvesting the whole plot; days to heading (DH) as the number of days from 1 January to when 50% of the spikes have emerged on 50% of all stems (Pask et al., 2012); plant height (PH) after flowering, when plants have reached their maximum height; hectoliter weight (HLW) by weighing a 550-mL volume of grains; thousand kernel weight (TKW) taking three random samples of 200 whole grains (removing all aborted and broken grains); and number of grains (NG) was calculated from GY and TKW. All agronomic traits were analyzed and expressed for each wheat variety as absolute values and as the percentage of a long-term check variety (a widely grown variety in the country) grown in the same trial and year: “Artur Nick” and “Nogal” for SW and WW, respectively (normalized values). Utilizing Artur Nick and Nogal as reference lines for estimating genetic gain in both SW and WW presents potential limitations: (1) Model Variability: the models employed in calculating yield progress (as depicted in **Figures 1C, D**) and their corresponding equations might exhibit variations when different reference varieties are used. These variations can influence the slopes and statistical significance of the models, potentially impacting the accuracy of the assessment. (2) Temporal variability in disease resistance of check varieties: the analysis exclusively relies on data from “ non-treated” trials. In such trials, the use of check varieties to gauge susceptibility may introduce inaccuracies due to the varying impact of diseases. However, disease affected both check and test varieties simultaneously, as evidenced by the significant positive correlation between check variety yield and the average yield of all tested varieties over the years (*y* = 1.05*x* + 2.1, *R* ^2^ = 0.69, *p* < 0.0001 for Artur Nick and *y* = 0.95x + 70.9, *R* ^2^ = 0.74, *p* < 0.0001 for Nogal). These correlations indicated environmental consistency: over the 15-year period, the trials likely occurred in relatively consistent environmental conditions, including soil type, climate, and other environmental factors, and in non-treated trials, the impact of diseases and environmental stressors may have been relatively consistent across varieties, leading to a uniform performance pattern. These correlations indicate that both Artur Nick and Nogal are adequate check varieties to calculate yield progress of newly released varieties over the years. Weather variables were provided by the local meteorological services at “Servei Meteorològic de Catalunya” and collected from the reference weather stations for the cultivation sites: maximum temperature (TM), average temperature (TA), and minimum temperature (Tm) expressed in °C, precipitation per day (P), and global solar radiation per day (SR), in mm and MJ m^−2^, respectively. The annual averages of weather variables were calculated. Correlations in SW regions were calculated using data from 2007 to 2021, and for WW regions, between 2013 and 2021 (according to weather data availability in each location).

**Table 1:** Long-term weather data for experimental trial locations of spring wheat (SW) and winter wheat (WW) in Catalonia: from 2007 to 2021 for La Tallada, Lleida, and Vilobí d’Onyar, from 2013 to 2021 for Solsona and Vic.

Location	Growth habit	W 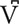	J	F	M	A	M	J	J	O	N	D	Year
**La Tallada**	SW	TM^˥^	14	15	17	19	23	27	30	22	17	15	21
		TA^±^	7	8	10	13	17	21	24	16	11	8	15
		Tm^‡^	1	2	4	7	11	15	17	11	6	2	9
		P^‖^	48	38	58	73	50	29	34	80	80	29	602
		SR^¶^	7	10	14	18	22	24	24	11	7	6	15
**Lleida**	SW	TM	10	14	17	20	25	30	32	22	15	10	21
		TA	5	7	10	13	17	22	24	15	9	5	14
		Tm	1	1	4	7	10	14	17	9	4	1	8
		P	27	18	32	51	39	30	27	39	42	16	364
		SR	6	11	16	20	25	27	27	13	8	5	17
**Vic**	WW	TM	10	13	16	18	23	28	31	21	14	11	20
		TA	4	6	9	11	15	20	23	14	8	4	13
		Tm	-1	0	2	5	8	13	15	9	3	0	7
		P	38	30	39	72	69	63	54	66	64	15	668
		SR											
**Solsona**	WW	TM	10	12	15	18	22	28	32	21	14	11	20
		TA	4	6	8	11	14	19	23	14	8	5	13
		Tm	0	1	2	5	7	12	15	8	3	0	7
		P	38	33	54	76	59	45	38	79	80	11	600
		SR	8	11	16	19	23	25	26	13	8	7	17
**Vilobí d’Onyar**	WW	TM	14	15	17	20	24	28	31	23	18	15	22
		TA	6	7	9	12	16	20	23	15	10	6	14
		Tm	-1	0	2	5	8	12	15	8	3	0	7
		P	43	38	58	71	66	48	35	74	83	25	645
		SR											

W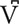, weather variables; TM^˥^, average maximum temperature (°C); TA^±^, average mean temperature (°C); Tm^‡^, average minimum temperature (°C); P^‖^, average of cumulative precipitation (mm); SR^¶^, average solar radiation (MJ m^−2^).

### 2.2 Statistical analysis

For each trial and season, the effects between varieties were tested using an analysis of variance (ANOVA). Absolute and normalized trait value means were obtained by averaging all the varieties per trial, year, and repetitions. Simple regression analysis was performed between (a) annual and monthly weather variable means and years; (b) absolute trait means and years, normalized trait means and years, and among each other; and (c) absolute trait means and annual and monthly weather variables. Regression analyses were performed using absolute and normalized trait means.

Linear, quadratic, and cubic fits were tested using absolute and normalized means against the years. Regression analyses were conducted for 2007–2021. The slope, observed through regression equations, was used to determine the estimated rate of genetic gain, expressed as the percentage yield (or other agronomic traits) above the check varieties per year. Slopes with probability levels < 0.05 were considered statistically significant, as well as *p*-values of 0.05 < *p* < 0.10. The statistical software package SAS-JMP Pro 16 (SAS Institute Inc., Cary, NC, USA, 1989–2019) was used to perform all reported statistical analyses. Graphs reporting the correlations and regression equations were plotted using Microsoft Excel 365 (Version 2304, Redmond, WA, USA).

## 3 Results

### 3.1 Climate change characterization in the major SW and WW growing regions of Catalonia

SW is grown in locations with higher average minimum temperatures (La Tallada and Lleida with yearly Tm of 9°C and 8°C, as shown in **Table 1**) whereas WW is grown in cooler locations (Vic, Solsona, and Vilobí d’Onyar) with yearly Tm of 7°C. The long-term average cumulative annual precipitation was higher than 600 mm at most locations, except for Lleida (364 mm) (**Table 1**), where SW is grown with irrigation. January to March were the coolest months with temperatures frequently lower than 0 in all three WW regions (**Table 1**). June and July represented the hottest months at all locations, with maximum temperatures above 30°C (**Table 1**).

Temperature in the main SW and WW locations of Catalonia (since 1990) exhibited an overall increase with a stable linear rate of 0.042-0.045°C per year, respectively, representing a total increase of 1.34°C in La Tallada in the past 32 years (**Supplementary Table 2**). Historical weather data analysis indicated significant changes in the SW area, with maximum (TM) and average temperatures (TA) increasing at rates of 0.086°C and 0.050°C per year, respectively. However, significant weather changes for WW regions have not been found (only nine years of averaged data are available; **Table 2**). Moreover, analysis per location indicated consistent increases of average and maximum temperatures, for both winter and spring cultivated area: annually, the TA in Vilobí increased at a rate of 0.042, TM in La Tallada at 0.042, and in Lleida at 0.043°C, in the last 23, 32, and 25 years, respectively (**Supplementary Table 2**). For SW, July and December temperatures were significantly increased: for TM at a rate of 0.170°C and 0.114°C, and for TA at a rate of 0.102 and 0.104 per year, respectively. Moreover, May P decreased at −2.41 mm per year. For WW, a significant increase in February TM was reported at a rate of 0.547°C per year. October TA and Tm decreased significantly at −0.281 and −0.356°C per year, respectively.

**Table 2:** Significant historical changes of annual and monthly weather variables in spring wheat (SW) (La Tallada and Lleida) and winter wheat (WW) locations (Vic, Solsona, and Vilobí d’Onyar) in Catalonia, Spain.

Growth habit	W 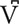	Regression equation	*R* ^2^	*p*-value	Years
SW	TM^˥^	*y* = 0.086Year−152.8	0.47	0.0048	15
	TA^±^	*y* = 0.050Year−86.9	0.34	0.0218	15
	SR^¶^	*y* = 0.043Year−71.3	0.20	***0.0926***	15
	May P^‖^	*y* = − 2.41Year+4,905.7	0.20	***0.0969***	15
	Jul TM	*y* = 0.170Year−310.4	0.32	0.0274	15
	Jul TA	*y* = 0.102Year−182.4	0.19	***0.0983***	15
	Dec TM	*y* = 0.114Year−216.5	0.25	***0.0542***	15
	Dec TA	*y* = 0.104Year−202.7	0.22	***0.0723***	15
WW	Feb TM	*y* = 0.547Year−1,089.7	0.38	***0.0740***	9
	Oct TA	*y* = − 0.281Year+581.0	0.40	***0.0634***	9
	Oct Tm	*y* = − 0.356Year+726.1	0.53	0.0247	9

W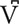, weather variables; TM^˥^, annual average maximum temperature (°C); TA^±^, annual average mean temperature (°C); Tm^‡^, annual average minimum temperature (°C); P^‖^, annual average of cumulative precipitation (mm); SR^¶^, annual average solar radiation (MJ m^−2^). Weather variables with significant changes over time are shown with regression equations, *R* ^2^, and probability values (*p* < 0.05, in bold with 0.05 < *p* < 0.10). The number of years (years) with available meteorological stations close to the wheat growing regions are shown.

### 3.2 Historical wheat grain yield progress in Catalonia and associated traits

To evaluate the GY progress associated with wheat breeding (for SW and WW varieties), the GY variation of new varieties released annually was calculated against the check varieties (normalized GY, **Figure 1**).

SW GY was significantly higher than WW by more than 1 T ha^−1^ (*p* < 0.0001; **Figures 1A, B**). Moreover, when considering each WW location, yield progress was significant in Vic and Solsona (*y* = 1.08*x* − 2068.9, *R* ^2^ = 0.23, *p* < 0.0796 for Vic and *y* = 1.46 *x* − 2,845.1, *R* ^2^ = 0.25, *p* < 0.0682). However, SW GY progress due to breeding has been stagnant for the past 15 years, as indicated by a nonsignificant linear regression across time (**Figure 1C**) and nonsignificant yield progress by location (*p*-value = 0.4 and 0.3 for La Tallada and Lleida, respectively). Moreover, the quadratic and cubic fits were not significant for SW. With regard to WW, the absolute GY changes in the rainfed WW regions of Catalonia were stagnant (**Figure 1B**). However, normalized GY showed a significant positive trend with significant linear, quadratic, and cubic fits (**Figure 1D**; **Supplementary Figure 1D**). To contrast this regional information, historical FAO GY data were also analyzed (including bread and durum wheat grain yields in Spain), showing nonsignificant yield progress in Spain (**Supplementary Figure 2**).

Average values of the analyzed traits for both SW and WW are shown in **Table 3**. GY, PH, HLW, TKW, and NG average values are higher for spring varieties. Simple linear regressions for normalized GY and traits across time indicated that in SW, PH decreased (at a −0.3 cm year^−1^ rate), whereas in WW, both HLW and NG increased over time (**Table 4**, see also results by location in **Supplementary Table 3**). The correlations of GY with HLW and NG were significant for both SW and WW (**Table 4**).

**Table 3:** Average, minimum (Min), maximum (Max), and standard deviation (Std. Dev) of grain yield (GY), days to heading (DH), date of heading (expressed as a date: “day –month”) plant height (PH), hectoliter weight (HLW), thousand kernel weight (TKW), and number of grains (NG) in spring wheat (SW) and winter wheat (WW).

Trait	Average		Min		Max		Std. Dev	
Growth Habit	SW	WW	SW	WW	SW	WW	SW	WW
GY (T ha^−1^)	8.02	6.86	3.89	1.96	13.15	12.86	1.58	2.00
GDD (°C)	1,209	1,003	982	819	1,557	1,181	87	71
Date of heading	19–4	6–5	1–4	9–4	9–5	3–6		
PH (cm)	90.0	82.5	60.0	55.0	119.5	128.5	10.6	9.7
HLW (kg hL^−1^)	79.5	73.9	64.1	50.5	88.3	87.3	3.9	5.4
TKW (g)	40.1	38.2	24.0	19.3	59.8	56.8	6.3	6.4
NG (grains m^−2^)	20,443.5	17,794.6	8,207.3	5,378.4	35,822.8	32,598.0	4,781.3	5,039.3

SW area included average data collected at La Tallada and Lleida; WW included average data collected at Vic, Solsona, and Vilobí d’Onyar.

**Table 4:** Significant (*p* < 0.10) correlations between normalized traits (% of check varieties “Artur Nick” and “Nogal” in spring and winter wheat, respectively), including grain yield (GY), days to heading (DH), plant height (PH), hectoliter weight (HLW), thousand kernel weight (TKW), and numbers of grains (NG) for both spring (SW) and winter wheat (WW) across time (years) and with GY.

Normalized trait or time	Growth habit	Normalized trait	Correlation	% norm change/year	abs change/year	*R* ^2^	*p*-value
Years	SW	GY	0.03	0.05%	0.004 T ha^−1^	0.00	0.9083
(15)		DH	0.19	0.03%	0.042 days	0.04	0.4998
		HLW	−0.10	−0.05%	−0.043 kg/hL^-1^	0.01	0.7147
		TKW	−0.40	−0.36%	−0.098 g/1,000 seeds	0.16	0.1383
		PH	−0.45	−0.32%	−0.292 cm	0.20	***0.0952***
		NG	−0.04	−0.06%	12.59 grains m^2^	0.00	0.8750
	WW	GY	0.60	1.31%	0.086 T ha^−1^	0.36	0.0171
		DH	0.33	0.04%	0.080 days	0.11	0.2261
		HLW	0.51	0.23%	0.173 kg/hL	0.26	***0.0513***
		TKW	0.25	0.36%	0.271 g/1,000 seeds	0.06	0.3646
		NG	0.54	1.04%	184.1 grains m^2^	0.29	0.0381
GY	SW	HLW	0.53			0.28	0.0426
		NG	0.78			0.61	0.0006
	WW	HLW	0.74			0.55	0.0014
		NG	0.83			0.69	0.0001

The colors reported in the table indicate the sign of correlations (Pearson values), either positive or negative, with shades of green and red, respectively; Non significant correlations are shown in grey. Probability values are shown in bold with 0.05 < p < 0.10.

### 3.3 Weather impact on historical wheat yield and associated agronomic traits

The means of absolute traits and annual weather variables were used in correlation analysis (**Table 5**). The HLW for SW and the DH for WW decreased over time at a rate of −0.382 and −0.815 per year, respectively (**Table 5**). In addition, DH was negatively correlated with Tm and TA in SW and WW, respectively. Moreover, in SW, HLW was negatively correlated with TM and TA. In WW, HLW negatively correlated with Tm and cumulative annual precipitation (P) (**Table 5**; **Supplementary Figure 3**). Regarding the correlations among traits for absolute values (indicative of the environmental and agronomic effects), positive correlations were observed in SW of yield with DH, PH, and NG. Moreover, significant positive correlations were depicted in WW for GY with PH and NG (**Supplementary Table 4**).

**Table 5:** Significant correlations (*p* < 0.10) of agronomic traits with time (Year) and between traits and annual weather variables, for spring wheat (SW) and winter wheat (WW).

Trait or time	Growth habit	WV* or Trait	Regression equation	Slope (units)	SE^~^	*R* ^2^	*p*-value	Years
**Year**	SW	HLW	*y* = − 0.382Year + 849.6	−0.382 (kg hL^−1^)	0.11	0.48	0.0041	15
	WW	DH	*y* = − 0.815Year + 1845.9	−0.815 (days)	0.29	0.27	0.0153	15
**HLW**	SW	TM	*y* = − 1.932TM + 120.6	−1.932 (kg hL^−1^)	1.09	0.19	***0.0995***	15
		TA	*y* = − 3.129TA + 125.5	−3.129 (kg hL^−1^)	1.55	0.23	***0.0647***	15
	WW	Tm	*y* = − 6.977Tm + 121.1	−6.977 (kg hL^−1^)	2.41	0.55	0.0229	9
		P	*y* = − 5.728P + 84.3	−5.728 (kg hL^−1^)	1.51	0.67	0.0069	9
**DH**	SW	Tm	*y* = − 8.145Tm + 196.1	−8.145 (days)	3.47	0.30	0.0355	15
	WW	TA	*y* = − 12.490TA + 305.7	−12.490 (days)	3.72	0.62	0.0122	9

* , weather variables; ~, standard error. HLW, Hectoliter weight; and DH, days to heading; Tm, minimum temperature; TA, average temperature; TM, maximum temperature and P, rainfall. Probability values in bold with 0.05 < p < 0.10.

To better understand the impact of weather variables, a correlation analysis was performed between agronomic traits and monthly weather variables (**Table 6**). The overall analysis indicated that agronomic traits were particularly influenced by weather in February and May (**Table 6** shows significant correlations with various agronomic traits). February temperatures negatively affected DH in both SW and WW. However, the May temperatures were negatively correlated with PH, HLW, and TKW. For SW, a negative correlation between GY and Tm in January and April and between GY and solar radiation (SR) in June and November were observed. For WW, GY was negatively correlated with TA and Tm in October (**Table 6**). The GY components showed negative correlations for both SW and WW with rainfall in January and June (for WW, also moderately in December), affecting HLW. In addition, for WW, a negative effect of Tm in May, Tm and TA in April, and TM in October were observed. However, this effect on the HLW was positive for Tm, TA, and TM in November. Regarding TKW, both SW and WW were negatively affected by temperature during spring. In SW, TA and Tm were negatively correlated with TKW in May. Moreover, at WW, the Tm was negatively correlated with TKW in May. Finally, the NG for SW was positively correlated with Tm and TA in May and with Tm in June.

**Table 6:** Correlations of monthly weather variable means, including maximum temperature (TM), average temperature (TA), minimum temperature (Tm), solar radiation (SR), and rainfall (P) per month, from January to December with trait means of grain yield (GY), days to heading (DH), plant height (PH), hectoliter weight (HLW), thousand kernel weight (TKW), and number of grains (NG) shown.

Trait	Growth habit	N. of yr	Jan	Feb	Mar	Apr	May	Jun	Jul	Oct	Nov	Dec
GY	SW	15	Tm			Tm*		SR*			SR*	
	WW	9								TA		
										Tm		
DH	SW	15		TM								
				TA								
				Tm								
				SR*								
	WW	9		TM			TA*					TA
				TA			TM*					Tm
				Tm								P*
PH	SW	15		TM			TM*		TA*			
							Tm*		Tm			
							TA*		P*			
	WW	9								P*		
HLW	SW	15	P					P				
	WW	9	P			Tm*	Tm	P		TM*	TA	P*
						TA*					Tm	
											TM*	
TKW	SW	15					TA*		P			P*
							Tm*					SR*
	WW	9	TM*			TA*	Tm			TM		
						TM*				TA*		
NG	SW	15					Tm*	Tm*	P			
							TA*					
	WW	9		P*						P		

Only significant (*p*-value < 0.10) (0.05 < *p* < 0.10 indicated with “*”) correlations are indicated. “N. of yr” indicates the number of years included in the regressions. The colors reported in the figure indicate the type of correlation, if positive or negative, with shades of green and red, respectively. The reported results for spring wheat (SW) are data from 2007 and 2021, and those for winter wheat (WW) are data from 2013 to 2021.

## 4 Discussion

### 4.1 Recent climate change observations in Catalonia

Temperature in the main SW and WW locations of Catalonia (since 1990) exhibited an overall increase. This finding agrees with previous reports, indicating an increase of 0.050°C per year in Southwest Europe during the last 30 years and 0.055°C per year for the entire European continent (Twardosz et al., 2021). However, the timeframe (15 years) in which the genetic yield progress in Catalonia was evaluated showed higher rates of increase in mean annual TM (0.086°C per year) and TA (0.050°C per year), for the SW area. Moreover, the analysis of monthly weather data in the SW regions showed that May precipitation significantly decreased, and July and December temperatures increased. July TA and TM increased at rates of 0.102 and 0.170°C per year, which means a total increase in the past 15 years of 1.53°C and 2.55°C, respectively; similarly, TA and TM in December increased at rates of 0.104 and 0.114°C per year. In the WW regions, February TM increased at a substantial rate of 0.547°C per year and October TA and Tm decreased in the past 9 years. The frequency of exceptional warm months is increasing notably over the past 5 years (Skrzyńska and Twardosz, 2023), which is in accordance with the observation of this study for WW regions of Catalonia, Spain for February TM.

### 4.2 Is the wheat yield progress due to breeding sufficient to maintain the rate of yield increase in the Catalonian region?

The extensive body of literature on yield progress for SW and WW provides a comprehensive overview of the diverse range of annual growth rates, spanning from 0.5% to 1.6%, and encompassing various timeframes (Sayre et al., 1997; Zhou et al., 2007; Acreche et al., 2008; Sadras and Lawson, 2011; Green et al., 2012; Lopes et al., 2012; Sanchez-Garcia et al., 2013; Lo Valvo et al., 2018; Rife et al., 2019). These previous studies have assessed breeding advancements in crop yield by using historically cultivated varieties that have been widely grown in a specific region. These varieties are subjected to rigorous testing side by side, in replicated experimental trials, with the yields of each variety analyzed through regression analysis.

Another method to gauge yield progress involves post-registration trials, typically conducted by local agricultural services to aid farmers in selecting the most suitable varieties for their region. In these trials, all newly released wheat varieties (from the private and public sectors) are tested annually against a benchmark variety that is extensively cultivated in the area. Regression analysis is also applied in this context to measure yield progress accurately. However, it is essential to note that when evaluating genetic yield progress using historical post-registration trials, the yield calculations must be compared against the benchmark variety. This precaution ensures that changes in yield are attributed to genetic factors rather than fluctuations in agronomy or environmental conditions (e.g., Graybosch and Peterson, 2010; Crespo-Herrera et al., 2018 for further details on this methodology). In this study, the yield progress rates were computed by analyzing post-registration trials conducted locally in the Catalonia region every year (lead by the Institute of Agrifood Research and Technology, IRTA and the Ministry of Agriculture, Departament d’Acció Climàtica, Alimentació i Agenda Rural). Notably, Artur Nick and Nogal (benchmark SW and WW varieties) have consistently ranked among the top 10 varieties with the highest production of certified seed in Spain over the past 15 years (Ministry of Agriculture, Departament d’Acció Climàtica, Alimentació i Agenda Rural). Regressions of normalized GY in SW from 2007 to 2021 did not reveal a significant improvement in the newly released varieties compared to the check variety “Artur Nick “. In the context of SW, a notable trend has emerged over the past 15 years, marked by a significant reduction in plant height. On average, SW varieties have shown a gradual decline at a rate of 0.3 cm per year (compared to the check variety) with an average plant height (all varieties over 15 years) of 90 cm, which is already 2 cm below the average plant height of thecheck variety (“Arthur Nick” with 92 cm). The continual reduction in plant height, historically undertaken to enhance lodging tolerance, has now fallen below the critical threshold of 1 m as identified by Fischer (2007) for realizing the maximum yield potential. This decline in plant height raises concerns about potential constraints on grain formation, often referred to as “source limitations,” which may be impeding progress in yield enhancement in SW. Given these compelling findings, it is strongly recommended, particularly within the unique agricultural context of this region, to exercise caution when considering any further reductions in the height of SW new varieties. However, in WW, an increase of 1.31% per year was reported, compared to the check variety “Nogal” (**Figure 1D**). Therefore, the calculated genetic gain reported in this study for WW is still within the range of previous reports (from the 1950s onwards), though it also fitted a quadratic function suggesting recent stagnation. Moreover, in the WW regions of Catalonia, wheat yield progress was accompanied by increased HLW and NG over time (both expressed as percentages of the check variety), indicating that these two traits positively contributed to GY in the latest WW varieties released in the past 15 years. Grain HLW is a measure of grain density and size. Frequently, the number of grains per unit area increases simultaneously when selecting for grain yield in breeding programs. However, under certain conditions and germplasm, the genotypes with the highest yields are also those with the highest NG and grain size (Griffiths et al., 2015). Generally, increased GY has been achieved with stable or even reduced grain weight, evidencing higher levels of phenotypic plasticity in grain number in response to the environment (Sadras, 2007). The observations by Griffiths et al. (2015) are in accordance with the results presented here regarding yield, HLW, and NG progress over time in Catalonia, with more recent varieties showing the largest grains. Although historical progress in breeding has been clearly associated with grain number, other studies have highlighted the positive association between grain yield and grain size (Lopes et al., 2012; Sukumaran et al., 2018). These results together with other observations in the literature indicate that increased grain yield through boosting grain number and size is possible and the simultaneous improvement of these two traits has the potential to increase grain yields under rising temperatures.

Despite the positive linear yield progress due to breeding in WW, this genetic yield progress (**Figure 1D**) was not accompanied by an overall absolute yield increase in the region (**Figure 1B**). This result indicated that progress in WW yield due to breeding and variety improvement has not been sufficient to sustain the negative impacts of other factors, such as weather. Historical weather variables were analyzed to test this hypothesis.

### 4.3 Climate change and wheat breeding impact on productivity and associated traits

Absolute GY progress in Catalonia has been stagnant in both spring and WW between 2007 and 2021, which is in accordance with a previous analysis from 2001 and onwards in North and South Europe (Lopes, 2022), using FAO data (**Supplementary Figure 2**) and national data in the same timeframe analyzed in this study. The SW area, including La Tallada and Lleida, is characterized by increased water availability resulting from high rainfall frequency (in La Tallada) or supplementary irrigation (in Lleida) and milder overall average temperatures. However, the WW areas are rainfed and generally cooler during winter. Under these conditions, even if there were no significant negative correlations between weather and annual GY averages, weather variables were correlated with HLW and DH in both SW and WW. In SW, both maximum (TM) and average temperatures (TA) were negatively correlated with HLW; for each °C increase in TM (*p* < 0.0995) and TA (*p* < 0.0647), a 1.932 and 3.129 kg hL^-1^ decrease in HLW, were observed, respectively. In WW, Tm and P were correlated with HLW, and for each °C increase in Tm, a 6.977 kg hL^−1^ (*p* < 0.0229) decrease in HLW was observed. A significant negative correlation was also observed between precipitation and HLW, and this may result from the erratic annual distribution of precipitation (*p* < 0.0069; **Supplementary Figure 4**). Furthermore, in SW, a decrease in the HLW was reported, corresponding to an average total loss of 5.73 kg hL^−1^ from 2007-2021 (**Table 5**).

As mentioned above, DH has decreased over time, and this was correlated with weather variables. For each °C increase in Tm, a reduction of 8.2 days in DH was reported in SW, and in WW, for each °C increase in TA, a reduction of 12.5 days DH was observed. A temperature increase results in significant reduction on the time to flowering (Menzel et al., 2006) in 542 plant species (both wild and cultivate) in 21 European countries, showing that phenological phases advanced by up to 4.6 days per °C in spring and summer, for the period between 1971 and 2000. Here, the observed reductions in DH result frequently in smaller crops, lower biomass and photosynthesis, and decreased tillering capacity and yield (Asseng et al., 2015). Furthermore, the robust correlations observed between agronomic traits and temperatures from February to May substantiate the heightened influence of temperature during the “critical period” (Fischer, 1975) in wheat. This period denotes a growth stage (between stem elongation and the transition to reproductive growth) when the crop attains its greatest susceptibility to environmental stressors, especially those capable of affecting yield potential, and in the Catalonia region, the “critical period” occurs between February and May. Should the temperature continue to rise, it is expected that DH reductions will cause a decrease in yields. Currently, yields only stagnate; however, if temperatures continue to rise at the observed rates, it is expected that yields will eventually start to decrease. Increasing temperatures can act as a relevant limiting factor, forcing crops to close in advance of their cycle, and consequently reduce their yield potential (García et al., 2015; García et al., 2016; García et al., 2018). These results highlight the relevance of re-evaluating sowing dates and vernalization requirements to fit optimal weather conditions and growing two or more varieties on farms to buffer yields under an erratic distribution of precipitation and increasing temperature. There is a promising opportunity to investigate the advancement of the wheat planting schedule, the reduction of vernalization prerequisites, or the integration of earliness per se genes to expedite the wheat growth cycle, thereby mitigating the risk of encountering terminal heat stress. This avenue of research warrants thorough exploration in the future. The GY components also showed negative correlations with the average April and May temperatures, significantly affecting grain size (negative correlation with TKW and HLW; see also **Supplementary Figure 5**). Previous studies (using controlled growth conditions) indicated the negative effect of nighttime temperatures (>20°C) during the reproductive stage until maturity on grain size and yield (Prasad et al., 2008). The historical temperature increase observed under natural field conditions in the present study contributed to a decrease in grain size and has not yet resulted in grain yield reduction. However, if night and daytime temperatures continue to rise at the observed rates, a grain yield penalty will eventually be observed.

## 5 Concluding remarks

Recent historical data have brought to light a concerning trend in SW yields characterized by stagnation. This phenomenon is likely intertwined with the gradual decline in plant height over time, a factor that has pushed plant stature below the optimal threshold. This diminishing plant height has, in turn, led to reduced biomass and a compromised capacity for assimilation, potentially impacting grain formation. In the context of WW, recent historical records indicated significant positive yield progress, which was likely attributed to enhancements in both grain size and number. Moreover, the ongoing rise in temperature and unpredictable precipitation patterns have exerted a discernible and adverse influence on both SW and WW. These climatic variables have particularly affected two key traits: hectoliter weight and days to heading. Consequently, it is imperative that substantial breeding efforts are undertaken to adapt and optimize phenological traits to optimal sowing dates, maintain plant height, increase grain size and number in response to the unpredictability in temperature and precipitation distribution patterns. Simultaneously, within breeding programs, there should be a concerted focus on selecting for increased grain size and number, especially under conditions of high temperature and drought stress. This approach will be instrumental in safeguarding yield potential in emerging wheat varieties, ensuring they can thrive in the changing climate and meet the demand for sustainable crop production.

## Data availability statement

The original contributions presented in the study are included in the article/**Supplementary Material**. Further inquiries can be directed to the corresponding author.

## Author contributions

DG analyzed the data and wrote the manuscript. RS collected and processed the data and reviewed the manuscript. JS collected data and reviewed the manuscript. JB collected data and reviewed the manuscript. JD reviewed the manuscript. AG-R reviewed the manuscript. ML designed the study and prepared the manuscript. All authors contributed to the article and approved the submitted version.

## References

[B1] Acreche M. M. Briceño-Félix G. Sánchez J. A. M. Slafer G. A. (2008). Physiological bases of genetic gains in Mediterranean bread wheat yield in Spain. Eur. J. Agron. 28, 162–170. doi: 10.1016/j.eja.2007.07.001

[B2] Asseng S. Ewert F. Martre P. Rötter R. P. Lobell D. B. Cammarano D.. (2015). Rising temperatures reduce global wheat production. Nat. Clim Chang 5, 143–147. doi: 10.1038/nclimate2470

[B3] Crespo-Herrera L. A. Crossa J. Huerta-Espino J. Vargas M. Mondal S. Velu G.. (2018). Genetic gains for grain yield in cimmyt’s semi-arid wheat yield trials grown in suboptimal environments. Crop Sci. 58, 1890–1898. doi: 10.2135/cropsci2018.01.0017 33343013 PMC7691759

[B4] FAOSTAT (2021) Area harvested. Available at: https://www.fao.org/faostat/en/#data/QCL.

[B5] Fischer R. A. (1975). Yield potential in a dwarf spring wheat and the effect of shading. Crop Sci. 15, 607–613. doi: 10.2135/cropsci1975.0011183X001500050002x

[B6] Fischer R. A. (2007). Understanding the physiological basis of yield potential in wheat. J. Agric. Sci. 145, 99–113. doi: 10.1017/S0021859607006843

[B7] García G. A. Dreccer M. F. Miralles D. J. Serrago R. A. (2015). High night temperatures during grain number determination reduce wheat and barley grain yield: a field study. Glob Chang Biol. 21, 4153–4164. doi: 10.1111/gcb.13009 26111197

[B8] García G. A. Miralles D. J. Serrago R. A. Alzueta I. Huth N. Dreccer M. F. (2018). Warm nights in the Argentine Pampas: Modelling its impact on wheat and barley shows yield reductions. Agric. Syst. 162, 259–268. doi: 10.1016/j.agsy.2017.12.009

[B9] García G. A. Serrago R. A. Dreccer M. F. Miralles D. J. (2016). Post-anthesis warm nights reduce grain weight in field-grown wheat and barley. Field Crops Res. 195, 50–59. doi: 10.1016/j.fcr.2016.06.002

[B10] Graybosch R. A. Peterson C. J. (2010). Genetic improvement in winter wheat yields in the Great Plains of North America 1959-2008. Crop Sci. 50, 1882–1890. doi: 10.2135/cropsci2009.11.0685

[B11] Green A. J. Berger G. Griffey C. A. Pitman R. Thomason W. Balota M.. (2012). Genetic yield improvement in soft red winter wheat in the eastern United States from 1919 to 2009. Crop Sci. 52, 2097–2108. doi: 10.2135/cropsci2012.01.0026

[B12] Griffiths S. Wingen L. Pietragalla J. Garcia G. Hasan A. Miralles D.. (2015). Genetic dissection of grain size and grain number trade-offs in CIMMYT wheat germplasm. PloS One 10, e0118847. doi: 10.1371/journal.pone.0118847 25775191 PMC4361556

[B13] Heino M. Kinnunen P. Anderson W. Ray D. K. Puma M. J. Varis O.. (2023). Increased probability of hot and dry weather extremes during the growing season threatens global crop yields. Sci. Rep. 13 3583. doi: 10.1038/s41598-023-29378-2 36869041 PMC9984494

[B15] Lopes M. S. (2022). Will temperature and rainfall changes prevent yield progress in Europe? Food Energy Secur 11, e372. doi: 10.1002/fes3.372

[B16] Lopes M. S. Reynolds M. P. Manes Y. Singh R. P. Crossa J. Braun H. J. (2012). Genetic yield gains and changes in associated traits of CIMMYT spring bread wheat in a “Historic” set representing 30 years of breeding. Crop Sci. 52, 1123–1131. doi: 10.2135/cropsci2011.09.0467

[B14] Lo Valvo P. J. Miralles D. J. Serrago R. A. (2018). Genetic progress in Argentine bread wheat varieties released between 1918 and 2011: Changes in physiological and numerical yield components. Field Crops Res. 221, 314–321. doi: 10.1016/j.fcr.2017.08.014

[B17] Menzel A. Sparks T. Estrella N. Koch E. Aasa A. Ahas R.. (2006). European phenological response to climate change matches the warming pattern. Glob Chang Biol. 12, 1969–1976. doi: 10.1111/j.1365-2486.2006.01193.x

[B18] Pask A. Pietragalla J. Mullan D. Reynolds M. (2012). Physiological breeding II: A field guide to wheat phenotyping (Mexico, D.F: CIMMYT).

[B19] Pequeno D. N. L. Hernández-Ochoa I. M. Reynolds M. Sonder K. Moleromilan A. Robertson R. D.. (2021). Climate impact and adaptation to heat and drought stress of regional and global wheat production. Environ. Res. Lett. 16, 54070. doi: 10.1088/1748-9326/abd970

[B20] Prasad P. V. V. Pisipati S. R. Ristic Z. Bukovnik U. Fritz A. K. (2008). Impact of nighttime temperature on physiology and growth of spring wheat. Crop Sci. 48, 2372–2380. doi: 10.2135/cropsci2007.12.0717

[B21] Rife T. W. Graybosch R. A. Poland J. A. (2019). A field-based analysis of genetic improvement for grain yield in winter wheat cultivars developed in the US central plains from 1992 to 2014. Crop Sci. 59, 905–910. doi: 10.2135/cropsci2018.01.0073

[B22] Sadras V. O. (2007). Evolutionary aspects of the trade-off between seed size and number in crops. Field Crops Res. 100, 125–138. doi: 10.1016/j.fcr.2006.07.004

[B23] Sadras V. O. Lawson C. (2011). Genetic gain in yield and associated changes in phenotype, trait plasticity and competitive ability of South Australian wheat varieties released between 1958 and 2007. Crop Pasture Sci. 62, 533–549. doi: 10.1071/CP11060

[B24] Sanchez-Garcia M. Royo C. Aparicio N. Martín-Sánchez J. A. Álvaro F. (2013). Genetic improvement of bread wheat yield and associated traits in Spain during the 20th century. J. Agric. Sci. 151, 105–118. doi: 10.1017/S0021859612000330 23365469 PMC3518273

[B25] Sayre K. D. Rajaram S. Fischer R. A. (1997). Yield potential progress in short bread wheats in northwest Mexico. Crop Sci. 37, 36–42. doi: 10.2135/cropsci1997.0011183X003700010006x

[B26] Skrzyńska M. Twardosz R. (2023). Long-term changes in the frequency of exceptionally cold and warm months in Europe, (1831–2020). Int. J. Climatol. 43, 2339–2351. doi: 10.1002/joc.7978

[B27] Statistical Institute of Catalonia (2021) Superficie agrícola. Por productos. Provincias. Available at: https://www.idescat.cat/indicadors/?id=aec&n=15422&lang=es.

[B500] Sukumaran S. Lopes M. S. Dreisigacker S. Reynolds M. (2018). Genetic analysis of multi-environmental spring wheat trials identifies genomic regions for locus-specific trade-offs for grain weight and grain number. Theor. Appl. Genet. 131, 985–998. doi: 10.1007/s00122-017-3037-7 29218375

[B28] Twardosz R. Walanus A. Guzik I. (2021). Warming in europe: recent trends in annual and seasonal temperatures. Pure Appl. Geophys 178, 4021–4032. doi: 10.1007/s00024-021-02860-6

[B29] Zhou Y. Zhu H. Z. Cai S. B. He Z. H. Zhang X. K. Xia X. C.. (2007). Genetic improvement of grain yield and associated traits in the southern China winter wheat region: 1949 to 2000. Euphytica 157, 465–473. doi: 10.1007/s10681-007-9376-8

