# Supplementary material for: Impact of rising temperatures on historical wheat yield, phenology, and grain size in Catalonia
Source: Front Plant Sci. 2023 Oct 11;14:1245362. doi: 10.3389/fpls.2023.1245362 (PMC10641378; doi:10.3389/fpls.2023.1245362)
Supplement: Supplementary file 1 [file DataSheet_1.docx]

**
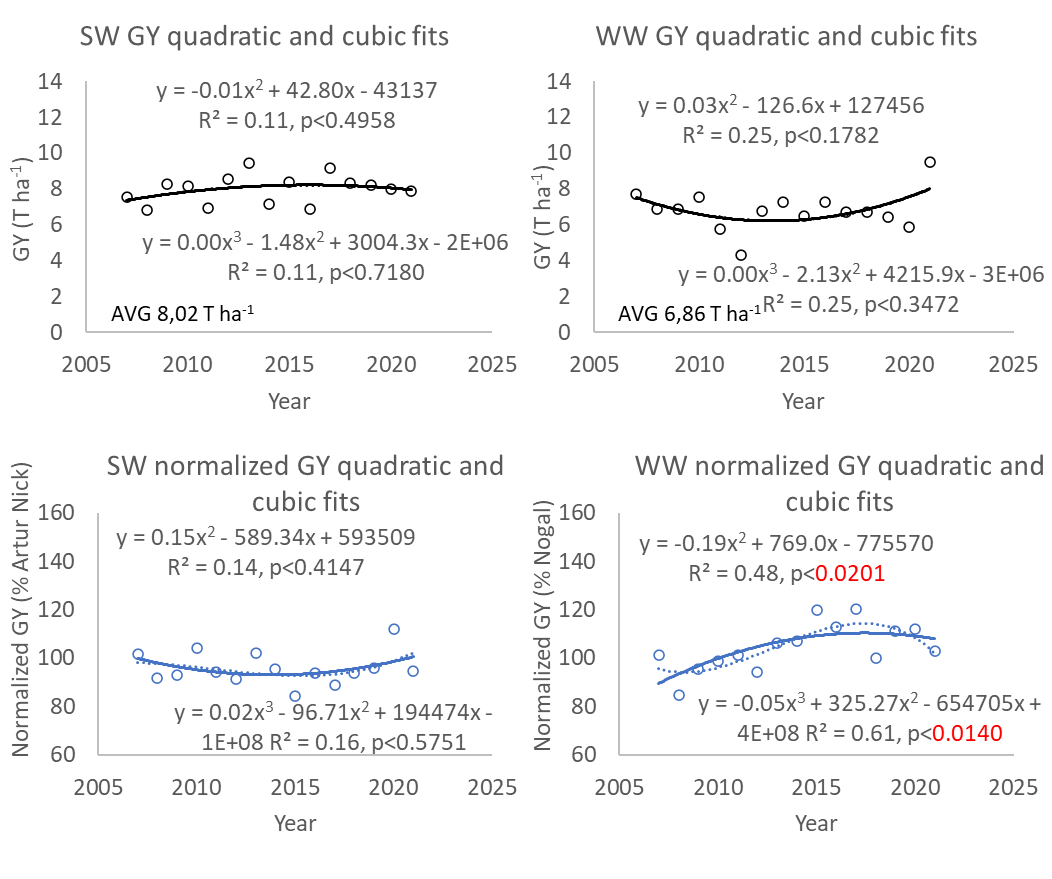
SUPPLEMENTARY MATERIALS**

**(D)**

**(C)**

**(B)**

**Supplementary Figure 1.** Fit regression models in spring wheat (SW) and winter wheat (WW) for GY progress in absolute values and as a percentage of GY variation (normalized GY) against check variety “Artur Nick” (SW) and “Nogal” (WW) in Catalonia for 2007‒2021. Quadratic (solid line) and cubic (round dotted line) non-linear functions are plotted, respectively: **(A)** for SW absolute GY, **(B)** for WW absolute GY, **(C)** for SW normalized GY and **(D)** for WW normalized GY. Means of all the varieties tested each year per each location are plotted and regression equations are shown with coefficients of determination (R^2^) and associated probability (in red significant p-values<0.05).

**(A)**


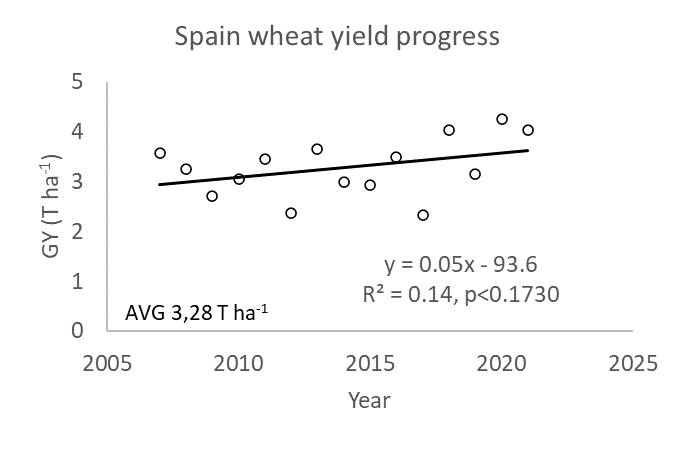


**Supplementary Figure 2.** Wheat yield progress in Spain (GY) in absolute values (expressed in T ha^-1^) for 2007‒2021 (FAOSTAT database). No significant correlation between GY and time (year) was reported. Regression equation is shown with coefficient of determination (R^2^) and associated probability. In Spain, winter wheat typically accounts for approximately 20% of the total cultivated area, while spring wheat makes up the remaining 80%. In Catalonia, on the other hand, the distribution differs significantly, with winter wheat covering approximately 90% of the total area, while spring wheat comprises the remaining 10%. (Official communication Departament d'Acció Climàtica, Alimentació i Agenda Rural, Spain).


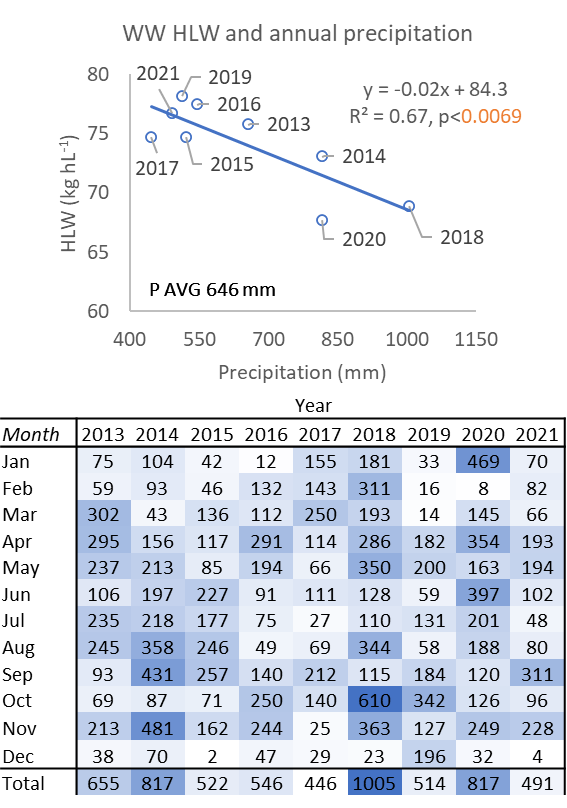


**(B)**

**(A)**

**Supplementary Figure 3.** Simple linear regression between hectoliter weight (HLW) and annual precipitation in winter wheat (WW) reporting every point of the plot labelled with the relative year in which the data were collected **(A)**; embedded table reporting the erratic distribution of precipitations in a year **(B)**. Means of all the varieties tested each year are plotted per each location and regression equation is shown with coefficients of determination (R^2^) and associated probability (in orange significant p-values<0.01).

**
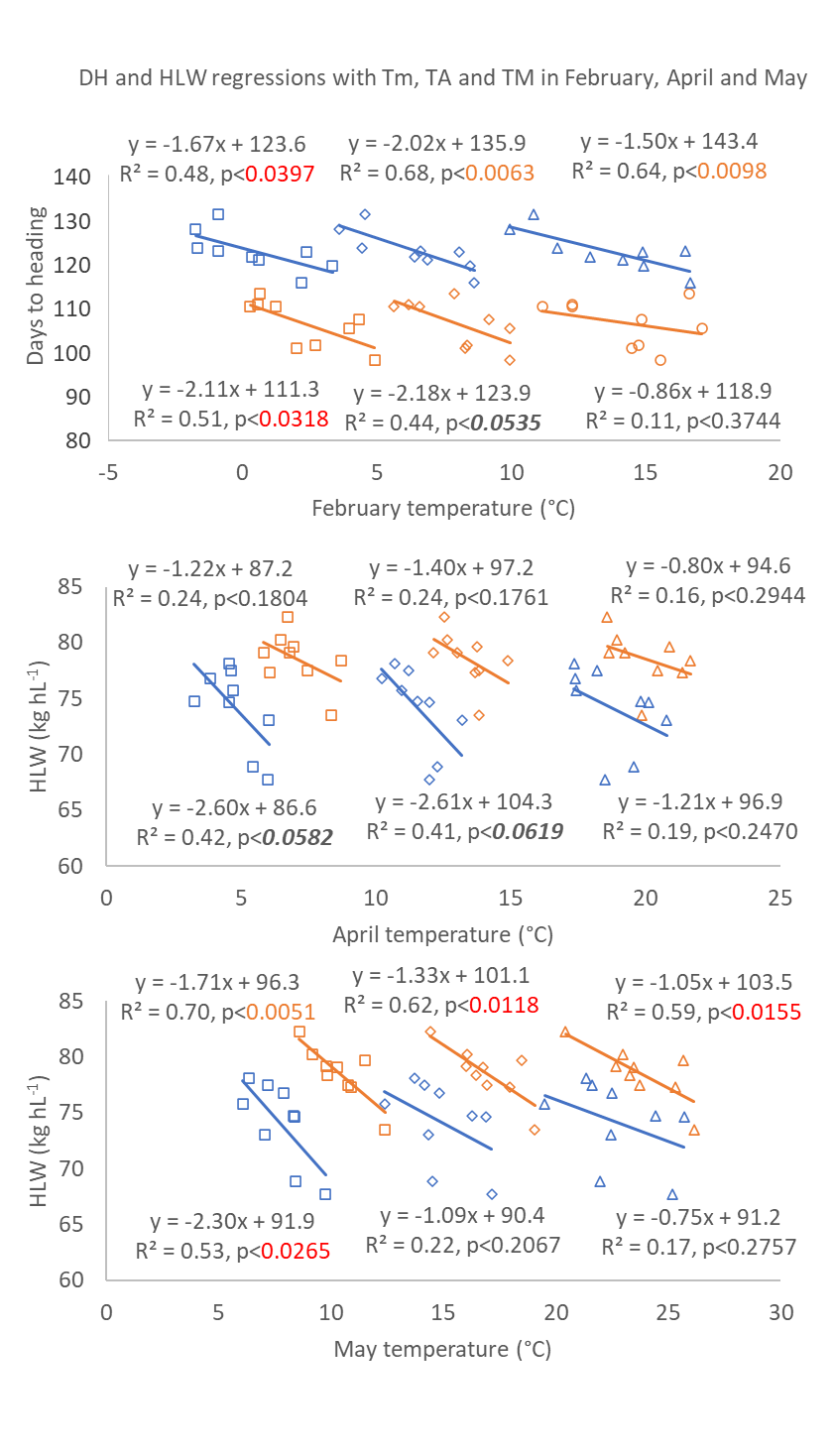
**

**(C)**

**(B)**

**(A)**

**Supplementary figure 4.** Simple linear regression of days to heading (DH) and hectoliter weight (HLW) with three temperature variables (from 2013 to 2021) in February **(A)**, April **(B)** and May **(C)**, which are minimum (Tm), average (TA) and maximum (TM), indicated by squares, rhombuses and triangles, respectively (blue and orange colored for winter and spring wheat, respectively). Means of all the varieties tested each year are plotted per each location and regression equations are shown with coefficients of determination (R^2^) and associated probability (in orange highly significant p-values<0.01, in red significant p<0.05, in bold and italic 0.05<p<0.10).

**Supplementary Table 1.** Annual count of spring and winter wheat varieties assessed in post-variety registration trials for genetic gain calculation (2007-2021).

|  | Nb Wheat Varieties | |
| --- | --- | --- |
| Year | Spring | Winter |
| 2007 | 17 | 29 |
| 2008 | 21 | 30 |
| 2009 | 19 | 33 |
| 2010 | 20 | 34 |
| 2011 | 20 | 30 |
| 2012 | 24 | 26 |
| 2013 | 25 | 45 |
| 2014 | 21 | 40 |
| 2015 | 19 | 46 |
| 2016 | 17 | 40 |
| 2017 | 22 | 44 |
| 2018 | 20 | 43 |
| 2019 | 19 | 38 |
| 2020 | 18 | 50 |
| 2021 | 19 | 45 |

**Supplementary Table 2.** Significant historical changes in the weather variables La Tallada and Lleida (spring wheat [SW]) and Vic, Solsona, and Vilobí d’Onyar (winter wheat [WW]) were observed in Catalonia, Spain. Weather variables showing significant changes over time are shown with regression equations, R^2^ and probability values (p<0.05, in bold and italic with 0.05<p<0.10). The number of years with available meteorological stations close to the wheat-growing regions (years) is shown.

| *Location* | *Years* | *Weather variable* | *Regression equation* | *p-value* |
| --- | --- | --- | --- | --- |
| La Tallada | 32 | TM^˥^ | y = 0.042Year-63.69 | 0.001 |
|  |  | TA^±^ | y = 0.021Year-27.61 | 0.0394 |
|  |  | SR^¶^ | y = 0.025Year-35.11 | 0.0088 |
| Lleida | 25 | TM^˥^ | y = 0.043Year-64.59 | 0.007 |
|  |  | SR^¶^ | y = 0.028Year-40.46 | 0.0179 |
| Vic | 10 | No significant correlation | | |
| Solsona | 9 | TM^˥^ | y = 0.150Year-282.11 | ***0.0578*** |
| Vilobí d’Onyar | 23 | TA^±^ | y = 0.042Year-71.38 | 0.006 |
|  |  | Tm^ǂ^ | y = 0.045Year-84.55 | 0.0183 |

TM^˥^, average maximum temperature (°C), TA±, average mean temperature (°C), Tm^ǂ^, average minimum temperature (Cº), SR^¶^, average global solar radiation (in MJ m^-2^).

**Supplementary Table 3** Significant (p<0.10) correlations between normalized traits (% of check varieties “Artur Nick” and “Nogal” in spring and winter wheat respectively), including grain yield (GY), days to heading (DH), plant height (PH), hectoliter weight (HLW), thousand kernel weight (TKW), and numbers of grains (NG) for experimental trial locations of spring (La Tallada and Lleida) and winter wheat (Vic, Solsona and Vilobì d’Onyar) across time (years) and with GY. The colors reported in the table indicate the type of correlation, if positive or negative, with shades of green and red, respectively.

| *Location* | *Normalized GY or time* | *Normalized Trait* | *Correlation* | *R2* | *p-value* | *Years of data* |
| --- | --- | --- | --- | --- | --- | --- |
| **La Tallada** | GY | HLW | 0,76 | 0,57 | 0,0045 | 12 |
|  |  | TKW | 0,64 | 0,41 | 0,0325 | 11 |
|  |  | NG | 0,94 | 0,89 | 0,0001 | 11 |
| **Lleida** | Year | PH | -0,59 | 0,34 | 0,0219 | 15 |
|  |  | TKW | -0,46 | 0,22 | ***0,0945*** | 14 |
|  | GY | DH | 0,44 | 0,20 | ***0,0986*** | 15 |
|  |  | HLW | 0,52 | 0,27 | 0,0453 | 15 |
| **Solsona** | Year | GY | 0,50 | 0,25 | ***0,0682*** | 14 |
|  |  | HLW | 0,49 | 0,24 | ***0,077*** | 14 |
|  | GY | HLW | 0,89 | 0,79 | 0,0001 | 14 |
|  |  | TKW | 0,82 | 0,67 | 0,0004 | 14 |
|  |  | NG | 0,72 | 0,51 | 0,0041 | 14 |
| **Vic** | Year | GY | 0,48 | 0,23 | ***0,0796*** | 14 |
|  |  | NG | 0,49 | 0,24 | ***0,0889*** | 13 |
|  | GY | HLW | 0,61 | 0,37 | 0,0201 | 14 |
|  |  | NG | 0,80 | 0,63 | 0,0012 | 13 |
| **Vilobí d'Onyar** | Year | HLW | 0,60 | 0,36 | 0,0175 | 15 |
|  |  | DH | 0,48 | 0,23 | ***0,0726*** | 15 |
|  | GY | DH | 0,58 | 0,33 | 0,0241 | 15 |
|  |  | NG | 0,93 | 0,87 | 0,0001 | 14 |

**Supplementary Table 4** Significant (p<0.10) correlations between absolute traits values, including grain yield (GY), days to heading (DH), plant height (PH), hectoliter weight (HLW), thousand kernel weight (TKW), and numbers of grains (NG) for both spring (SW) and winter wheat (WW) across time (years) and with GY.

| *Growth habit* | *Absolute GY or time* | *Absolute trait* | *Correlation* | *R2* | *p-value* | *Years of data* |
| --- | --- | --- | --- | --- | --- | --- |
| **Spring wheat** | GY | DH | 0,55 | 0,30 | 0,0328 | 15 |
|  |  | PH | 0,48 | 0,23 | ***0,0734*** | 15 |
|  |  | NG | 0,58 | 0,33 | 0,0247 | 15 |
| **Winter wheat** | GY | PH | 0,73 | 0,53 | 0,0021 | 15 |
|  |  | NG | 0,73 | 0,53 | 0,0021 | 15 |
